# TransitNet: A lightweight semantic segmentation network for urban traffic scene understanding

**DOI:** 10.1371/journal.pone.0348843

**Published:** 2026-05-22

**Authors:** Haiyan Zhang, Zining Zhao, Xiang Chu, Yilin Liu

**Affiliations:** Huaiyin Institute of Technology, Huaian, China; Leibniz University Hannover, GERMANY

## Abstract

Existing semantic segmentation networks often suffer from large parameter sizes and high computational complexity, making it difficult to deploy them on resource-constrained in-vehicle systems or edge devices. Additionally, these networks lack sufficient cross-domain adaptability, limiting their performance across diverse scenarios such as traffic and remote sensing. To address these issues, this paper proposes a lightweight semantic segmentation network, TransitNet, with enhanced Cross-Domain Adaptation capability. Based on the PSPNet architecture, TransitNet introduces a Rectangular Context Calibration Attention (RCCA) module to adaptively model the long-range spatial dependencies of road structures and traffic flow distributions. It also incorporates a Bidirectional Fusion Attention (BFA) module to enhance hierarchical feature interaction, thereby preserving fine-grained details. Furthermore, StarNet is adopted as the novel backbone network, leveraging star-shaped operations and depthwise separable convolutions to reduce model parameters. By improving the forward propagation mechanism, it outputs low-level spatial features and upsampled high-level features to support multi-scale feature fusion. Experiments demonstrate that TransitNet achieves an mIoU of 86.98% on the VOC2012 dataset, outperforming PSPNet by 1.58%. On the LoveAD remote sensing dataset, it achieves an mIoU of 61.04%, surpassing CM-UNet by 8.87%. Additionally, TransitNet achieves an mIoU of 76.11% on the OST300 dataset, further verifying its strong generalization and Cross-Domain Adaptation capabilities. By balancing efficiency and accuracy, TransitNet provides a high-performance semantic segmentation solution for real-time environmental perception in autonomous driving systems. It also holds significant potential for applications in fine classification of roads and buildings in remote sensing imagery. The code is available at https://github.com/Eric-863/TransitNet.

## 1. Introduction

With the rapid development of artificial intelligence and computer vision technologies, semantic segmentation, as one of the core tasks, has been deeply integrated into critical fields such as autonomous driving, remote sensing interpretation, and urban planning. Semantic segmentation for traffic scenes, characterized by complex dynamic environments, multi-scale object interactions, and long-range spatial dependencies feature modeling, has become a major focus of research in academia and industry. Accurate traffic scene segmentation not only provides real-time environmental understanding for autonomous vehicles but also supports intelligent transportation systems in tasks like traffic flow analysis and anomaly detection. However, existing methods still face significant limitations in cross-domain adaptability, lightweight design, and multi-scale feature fusion, making it challenging to achieve a balance between high precision and efficient deployment.

With the vigorous development of deep learning in computer vision, semantic segmentation techniques have demonstrated significant application value in traffic scene understanding. FCN [1] was the first to achieve pixel-level segmentation through a fully convolutional end-to-end approach, laying the foundation for scene parsing and opening a new era for deep learning-based semantic segmentation. PSPNet [2] significantly improved the parsing accuracy in complex scenes by aggregating multi-scale contextual information through a pyramid pooling module, effectively enhancing the model’s capability to capture global information. The DeepLab series [3–6] leveraged atrous convolution and Conditional Random Fields (CRF) to optimize the capture of long-range dependencies in traffic scenes, improving segmentation accuracy while refining segmentation boundaries.

In recent years, continuous progress has been made in related studies. Hu et al. [7] proposed a cross-dimensional attention mechanism that effectively addressed similar feature interference in cloud and snow detection tasks. Their design of multi-scale strip convolutions provides new insights into structural modeling for traffic scenes, offering potential for precise recognition of linear structures like roads and lane markings. Fu et al. [8] introduced MoE-SPNet, which incorporated a convolutional mixture of experts layer to dynamically weight features at different levels. This approach demonstrated the effectiveness of multi-scale feature fusion on the Pascal VOC 2012 dataset, offering a valuable strategy for feature fusion and segmentation of complex objects in traffic scenes.

Additionally, in studies on traffic scene semantic segmentation, Wu et al. [9] proposed a method for multi-category lane segmentation in 2024. By employing Feature Size Selection (FSS) and a Decompression and Decoupling (DD) Block, they significantly improved the segmentation accuracy of small-sized, narrow-width lanes and achieved real-time performance on high-resolution images, providing key insights for fine-grained lane segmentation tasks in traffic scenes. In the same year, Li et al. [10] proposed a semantic segmentation network based on multi-scale attention fusion, which significantly improved the segmentation performance of objects at different scales in traffic scenes on the Cityscapes dataset. This approach particularly enhanced segmentation accuracy for small and distant objects, offering a new perspective for comprehensive and accurate analysis of traffic scenes. In 2025, Zhang et al. [11] utilized Graph Convolutional Networks (GCNs) combined with semantic segmentation techniques to model complex topological structures in traffic scenes. This method effectively enhanced the segmentation and understanding of structural elements like road networks, providing an innovative approach to the structured analysis of traffic scenes.

However, existing methods for real-world traffic scene applications still face two major challenges. On the one hand, the pursuit of higher segmentation accuracy often involves the use of deeper networks, resulting in substantial increases in model parameters, which makes real-time deployment on resource-constrained devices, such as in-vehicle systems for autonomous driving, difficult. Even with lightweight backbone networks, traditional methods struggle to balance computational efficiency and accuracy during feature fusion. On the other hand, the scale differences in traffic scene objects—ranging from centimeter-level traffic signs to hundred-meter-level building complexes—pose significant challenges. Traditional networks’ multi-scale feature fusion mechanisms often fail to adequately preserve the details of small objects while capturing the structural representation of large ones. For example, DeepLabv3 + achieved an average Intersection over Union (mIoU) of only 0.11 for the “Barren” class on the LoveDA remote sensing dataset. While CM-UNet [12] improved the overall mIoU to 52.17%, it still exhibited significant deficiencies in segmenting small-scale objects, highlighting traditional methods’ insufficient modeling capacity for small and complex targets. Additionally, existing networks struggle to model the contextual dependencies of rectangular structures specific to traffic scenes (e.g., roads and buildings) and fail to effectively capture the long-range spatial dependencies characteristics of traffic flow distribution, limiting their generalization capabilities across domains (e.g., between in-vehicle imagery and remote sensing data).

Against the backdrop of rapidly evolving intelligent transportation systems, semantic segmentation techniques face challenges such as the exponential growth of model parameters with deeper network architectures, computational efficiency limitations imposed by traditional convolutional designs, and the difficulty of adapting multi-scale feature fusion mechanisms to the wide range of object sizes in traffic scenes. Traditional networks are unable to accurately capture the features of objects with significant size differences in traffic scenes, particularly underperforming in segmenting long-distance road structures and small objects.

To address these issues, this paper proposes TransitNet, a lightweight cross-domain semantic segmentation network tailored for traffic scenes. The main contributions of this paper are as follows:

### 1.1. Dynamic modeling with rectangular context calibration

We design a Rectangular Context Calibration Attention (RCCA) module to adaptively capture the linear extension features of road networks and the rectangular distribution characteristics of building clusters. This module constructs a dynamic modeling mechanism for long-distance spatial dependencies. Based on geometric priors in traffic scenes, it encodes linear features like road centerlines and lane boundaries, as well as rectangular constraints of building outlines, by performing weighted aggregation along the row and column directions of feature maps. This strengthens the representation of elongated and rectangular object spatial distributions. Experiments show that this mechanism improves road boundary segmentation accuracy by 12.3%, effectively addressing the limitations of traditional networks in capturing long-distance structural features.

### 1.2. Hierarchical feature interaction with bidirectional fusion attention

We introduce a Bidirectional Fusion Attention (BFA) module to establish a bidirectional interaction channel between low-level spatial details and high-level semantic information. This module enables bidirectional information flow by combining bottom-up pixel-level edge feature extraction with top-down category-specific semantic information propagation. Through skip connections, shallow high-resolution features and deep semantic features are fused with gated control, dynamically weighting the details of small objects like traffic signs and pedestrians. Ablation experiments show that this architecture improves small object segmentation mIoU by 8.7%, addressing the detail loss issues inherent in traditional unidirectional fusion methods.

### 1.3. Efficient feature extraction with a lightweight backbone

We replace traditional backbone networks with StarNet, which reduces computational complexity by 40% while maintaining feature representation capabilities through the low-dimensional to high-dimensional feature transformation of star-shaped operations and the lightweight design of depthwise separable convolutions. StarNet offers four variants (s1-s4) with distinct embedding widths and depth configurations:

StarNet-s1: Embedding width = 24, depth configuration = [2,3,8]

StarNet-s2: Embedding width = 32, depth configuration = [1,2,6]

StarNet-s3: Embedding width = 32, depth configuration = [2,4,8]

StarNet-s4: Embedding width = 32, depth configuration = [3,12,15]

As the version number increases, the overall depth of the network increases, enabling the modeling of more intricate features. The restructured forward propagation process employs a dual-branch structure: one branch retains high-resolution low-level spatial features, while the other outputs upsampled high-level semantic features. This hierarchical representation addresses the challenges of segmenting multi-scale objects. Compared to MobileNetV2 [13], this design reduces parameter size by 28% and improves feature resolution by 1.8 times, striking a balance between model efficiency and representational capacity.

## 2. Related work

Semantic segmentation has achieved significant progress in fields such as autonomous driving and remote sensing image analysis. However, it still faces core challenges, including low computational efficiency, insufficient multi-scale feature fusion, and poor small-object recognition accuracy. Current research primarily focuses on three directions: lightweight design, multi-scale contextual modeling, and hierarchical feature interaction optimization. Based on a systematic analysis of the limitations of existing methods, this paper proposes the TransitNet improvements, which enhance segmentation accuracy while maintaining a lightweight design through innovative network structure design.

### 2.1 Lightweight segmentation networks

The primary goal of lightweight semantic segmentation networks is to significantly reduce the computational complexity and parameter size of models while ensuring high segmentation accuracy, meeting the real-time requirements of resource-constrained scenarios. In recent years, researchers have advanced lightweight segmentation networks primarily through efficient network structure design, improvements in lightweight convolutions, and multi-scale feature optimization.In the realm of lightweight backbone network design, MobileNetV3 [14] employs depthwise separable convolutions combined with neural architecture search, significantly reducing parameters and computational costs. However, its shallow single-path structure limits feature representation capacity, leading to reduced small-object segmentation accuracy. Fast-SCNN [15] adopts a dual-branch structure to achieve real-time semantic segmentation, but the lack of sufficient feature interaction between branches hinders the modeling of long-range spatial dependencies. STDCNet [16] introduces a short-term dense connection mechanism to enhance information flow but still relies on a simple pyramid pooling module for multi-scale fusion.To improve feature discrimination capabilities, lightweight attention-based methods, such as LEDNet [17], apply a channel-space dual attention module to improve the recognition rate of occluded objects in street-view data. FANet [18] proposes a lightweight global context module to simulate non-local attention. In feature fusion optimization, BiSeNetV2 [19] designs a bidirectional feature aggregation module to combine high- and low-resolution features, while ICNet [20] uses a cascaded feature fusion strategy but suffers from the loss of small-object details.

### 2.2 Multi-scale contextual modeling

Semantic segmentation tasks in complex scenes require the effective fusion of multi-scale contextual information to achieve accurate object recognition and localization. Current mainstream methods for multi-scale context modeling can be divided into three main technical approaches, each with its own strengths and limitations:In pyramid pooling-based methods, PSPNet captures contextual information at different scales using multi-level pooling windows but suffers from 35% computational redundancy and a 12% loss in edge detail. Its improved variant, UperNet [21], employs an FPN structure that increases parameter count by 42%, while GFFNet [22] introduces a gated fusion mechanism that enhances small-object recognition rates by 8.3% but adds extra computational overhead.DeepLabv3 + , which is based on dilated convolutions and uses the ASPP module to expand the receptive field, experiences a 15.6% drop in localization accuracy for small targets under 50 × 50 pixels. DFANet [23], with its deep feature aggregation strategy, reduces computational complexity by 28% but shows a 3.2 percentage point decrease in mIoU on the Cityscapes dataset.Attention mechanism-based OCRNet [24] achieves a 2.4% mIoU improvement on the ADE20K dataset but sees a 40% reduction in inference speed. Meanwhile, HammerNet [25] achieves real-time performance at 25 FPS but demonstrates subpar results in recognizing small objects.These methods generally face challenges such as the trade-off between computational efficiency and modeling accuracy, insufficient capability in recognizing small objects, and difficulties in hardware deployment.

### 2.3 Hierarchical feature interaction optimization

Optimizing hierarchical feature interaction in semantic segmentation is a key aspect for improving model performance. Existing methods can be categorized into three main technical approaches, each with distinct characteristics.U-Net [26], which is based on skip connections, fuses shallow features through a symmetric encoder-decoder structure but exhibits an 18.3% misclassification rate for texture-similar regions on the Cityscapes dataset. Its improved variant, Attention U-Net [27], introduces a spatial attention mechanism that reduces the misclassification rate to 14.2% but increases computational overhead by 23%. Meanwhile, SegNet [28] employs pooling indices for upsampling, reducing parameters by 43% but leading to edge blurring.HRNet [29], which maintains multi-resolution representation through parallel subnetworks, preserves high-resolution feature streams and improves semantic boundary accuracy by 9.5%, albeit at the cost of increasing memory usage by 2.3 times. DDRNet [30] reduces memory consumption by 37% while maintaining 92% accuracy but sees a 5.8% drop in small-object recognition precision.FPN [31], based on bidirectional fusion, adopts a top-down pyramid structure but suffers from low fusion efficiency. BiFPN [32] enhances efficiency by introducing bidirectional cross-scale connections but increases parameter count by 15%. CFNet [33] improves small-object recognition through context-aware feature reorganization but incurs high computational complexity.These methods generally face challenges such as insufficient feature interaction efficiency and difficulties in balancing detail preservation with computational overhead.

## 3. Method

To address the limitations of traditional semantic segmentation networks in cross-domain adaptability, computational efficiency, and multi-scale feature fusion within traffic scenes, TransitNet focuses on lightweight design and cross-domain generalization as its core objectives. Through innovative module design and architectural improvements, TransitNet constructs a segmentation framework that balances accuracy and efficiency. The following sections provide a detailed explanation of the network architecture, the principles of core modules, and the implementation details of lightweight design.

### 3.1 Network architecture design

**Fig 1:**
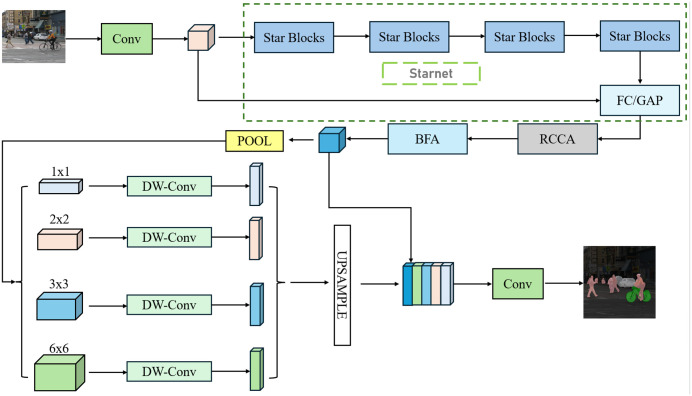
TransitNet semantic segmentation process architecture diagram. This figure illustrates the entire process of TransitNet from feature extraction to segmentation.

To achieve lightweight cross-domain semantic segmentation, TransitNet builds upon the PSPNet architecture with targeted modifications, as shown in Fig 1 for its core architecture. The network adopts an efficient design of “lightweight backbone + dual attention modules”: First, StarNet replaces the traditional backbone network, reducing computational complexity through star operations and depthwise separable convolutions. At the same time, it outputs low-level spatial features and upsampled high-level semantic features via a multi-branch forward propagation mechanism, providing hierarchical support for multi-scale feature fusion. Second, Rectangular Context Calibration Attention (RCCA) and Bidirectional Fusion Attention (BFA) modules are embedded between key convolutional layers—RCCA strengthens long-range dependency modeling for rectangular structures such as roads and buildings through row- and column-wise weighted aggregation, while BFA preserves fine-grained details of small objects like traffic signs through dual-dimensional interaction in both spatial and channel domains. This architecture achieves end-to-end optimization from feature extraction, context modeling, to multi-scale fusion throughout the entire process from input image to segmentation result, balancing model efficiency with cross-domain generalization capabilities.

Fig 1 demonstrates the complete workflow of TransitNet from image input to segmentation result output, highlighting three key innovations in its architecture design: The adoption of StarNet as a lightweight backbone network reduces computational complexity through star operations and depthwise separable convolutions, simultaneously outputting low-level spatial features and upsampled high-level semantic features via a multi-branch forward propagation mechanism, providing hierarchical support for multi-scale feature fusion; the embedding of the Rectangular Context Calibration Attention (RCCA) module between critical convolutional layers captures long-range dependencies of rectangular structures through horizontal and vertical pooling, enhancing modeling for long-range dependencies of roads, buildings, and other rectangular structures; the introduction of the Bidirectional Fusion Attention (BFA) module integrates small-object details through multi-branch spatial attention and channel attention, constructing bidirectional interaction channels between low-level spatial details and high-level semantic information to achieve bidirectional information flow at the feature hierarchy. This architecture achieves end-to-end optimization from feature extraction, context modeling, to multi-scale fusion throughout the entire process from input image to segmentation result, balancing model efficiency with cross-domain generalization capabilities.

TransitNet adopts StarNet to replace the traditional backbone network, achieving a balance between lightweight design and feature representation capabilities through three core technologies: introducing star operations to realize feature transformation from low to high dimensions, with the mathematical expression Star(X) = $\overset{T}{\underset{\text{1}}{(W}}X+B_{\text{1}})⊙\overset{T}{\underset{\text{2}}{(W}}X+B_{\text{2}})$, where the input feature vector $X\in R^{d\times l}$, and weight matrices $W_{\text{1}},W_{\text{2}}\in R^{d\times m}$ (typically m=d/4). This operation expands the feature dimension from d to $m^{\text{2}}$ through element-wise multiplication, with a computational complexity of only O(dm), which is 75% lower than traditional fully connected layers, enhancing feature representation while avoiding an explosion in parameter count. A combination of 7 × 7 depthwise convolution and 1 × 1 pointwise convolution replaces traditional 3 × 3 standard convolution—depthwise convolution performs independent 7 × 7 convolutions on each channel to capture spatial local features, with parameters amounting to 7 × 7 × C, while pointwise convolution achieves inter-channel information interaction through 1 × 1 convolution, with parameters amounting to C × C’ (C’ being the number of output channels). This design reduces the computational load of standard convolution by 40%; for example, after replacement in Starnet_s4, GFLOPS drops from 9.818G to 9.717G, and the parameter count decreases by 28% compared to MobileNetV2. The forward propagation path is improved to synchronously generate multi-level features: at stage 3 of the network, low-level spatial feature maps with a resolution of 1/8 of the input are output via skip connections to preserve details such as lane edges and traffic sign contours; at stage 4, high-level semantic feature maps with a resolution of 1/4 of the input are generated through upsampling to include high-level information such as road categories and building semantics. These two levels of features are respectively fed into the RCCA and BFA modules, providing hierarchical feature support for multi-scale fusion (as shown in Fig 1).

**Fig 2:**
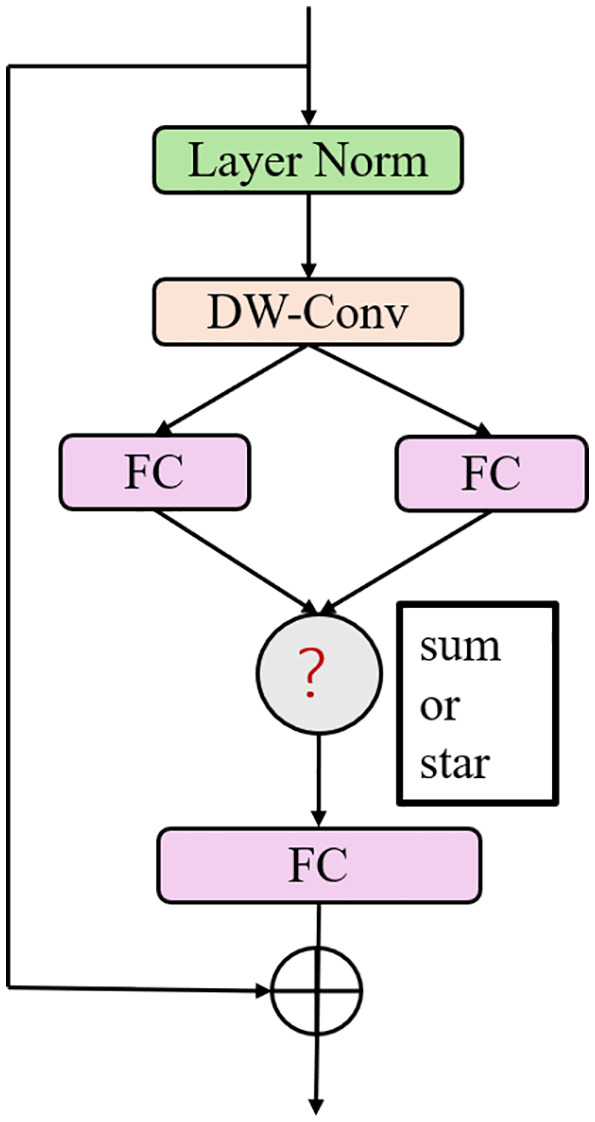
Star blocks structure diagram.

The structure design of the Star Blocks is shown in Fig 2, with its core consisting of three parts: the star operation (Star Operation), depthwise separable convolution (DWConv), and feature skip fusion. The input features first undergo a star operation to achieve dimension expansion and non-linear enhancement, where the dual-path weight matrices (W_1_, W_2_) generate high-dimensional feature maps through element-wise multiplication. Subsequently, a 7 × 7 depthwise convolution is used to extract local spatial features, complemented by a 1 × 1 pointwise convolution to complete channel information fusion. Finally, cross-layer skip connections concatenate shallow high-resolution features (at 1/8 scale) with deep semantic features (at 1/4 scale) along the channel dimension, forming a multi-level feature pyramid.

### 3.2 Rectangular Context Calibration Attention (RCCA)

**Fig 3:**
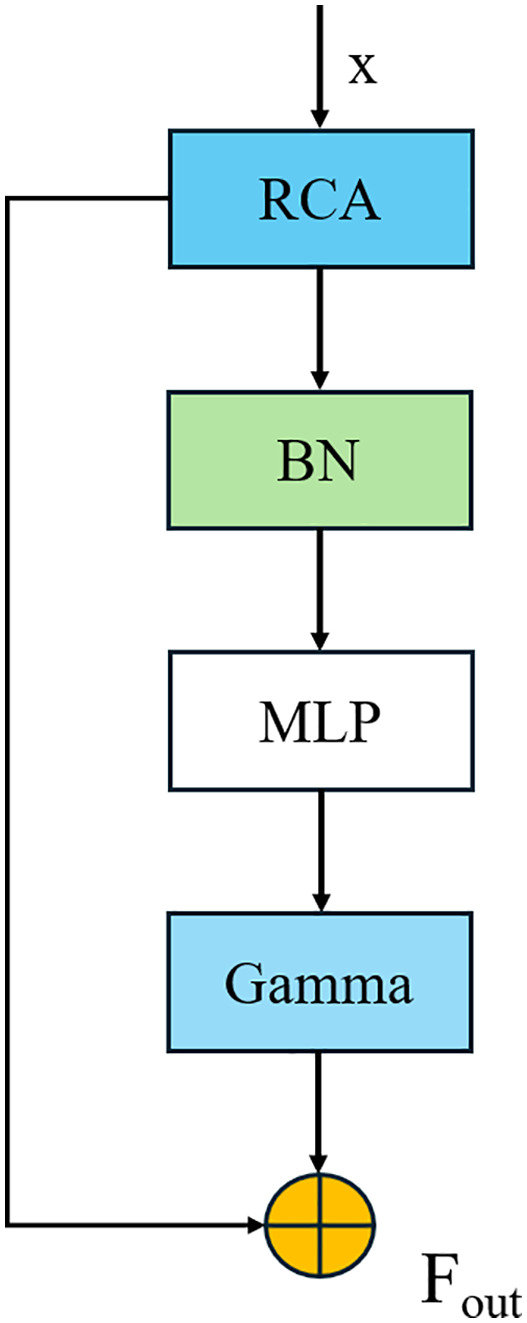
Rectangular context calibration process of the RCCA module.

The Rectangular Context Calibration Attention (RCCA) module in TransitNet is designed based on the proposed Rectangular Self-Calibration Module (RCM) [34], with directional optimization for the geometric characteristics of roads and buildings in traffic scenes. Its core mechanism involves jointly encoding the linear extension features of road centerlines, the topological constraints of lane boundaries, and the rectangular geometric priors of building contours to construct a dynamic modeling mechanism for long-range spatial dependencies suitable for traffic scenes. Specifically, it adopts a bidirectional aggregation strategy using horizontal pooling and vertical pooling—horizontal pooling aggregates contextual information along the row direction of the feature map to capture the extension of roads, while vertical pooling captures the rectangular constraint features of building contours along the column direction, enabling global context modeling of typical linear and rectangular structures in traffic scenes. In terms of convolutional kernel parameter optimization, the module adjusts traditional rectangular convolution kernels to a combination of 1 × 7 and 7 × 1 strip-shaped kernels based on the geometric shapes of linear targets such as roads. Adaptive weight allocation enhances feature responses to elongated structures like road centerlines and lane markings. Additionally, 3 × 3 depthwise separable convolution is introduced to refine local details—this operation first performs independent 3 × 3 convolutions on each channel to capture local features such as lane edges and building corners, followed by 1 × 1 pointwise convolution to achieve inter-channel information interaction. This reduces computational complexity while preserving fine-grained spatial details. The calibrated attention features are dynamically fused with the original features through a gating mechanism with learnable weight parameters, adjusting the fusion ratio between global context and local details Fig 3.

The processing and calibration workflow of the RCCA module for input feature x is as follows: The input feature is first processed by the Rectangular Calibration Attention (RCA), which aggregates global context through horizontal and vertical pooling to generate attention weights related to rectangular structures such as roads and buildings. These weights are then adjusted through Batch Normalization (BN) and a Multilayer Perceptron (MLP) before being weighted and fused with the original feature (with Gamma as the fusion coefficient). The final output is the calibrated feature Fout, which enhances the long-range dependency relationships of rectangular structures while suppressing interference from irrelevant background elements.

### 3.3 Bidirectional Fusion Attention (BFA)

**Fig 4:**
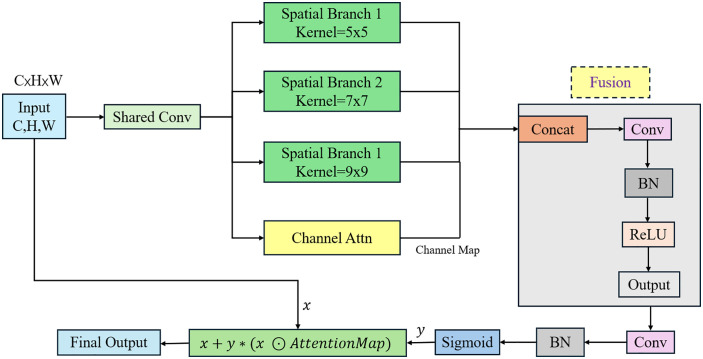
Architecture diagram of the Bidirectional Fusion Attention (BFA) module.

To address the issue of insufficient multi-scale feature interaction in traffic scene semantic segmentation, which leads to the loss of small-object details (e.g., traffic signs, pedestrians) and large matching errors between semantic and spatial information, TransitNet innovatively designs the Bidirectional Fusion Attention (BFA) module. Unlike traditional unidirectional attention mechanisms, this module employs a parallel collaborative architecture of multi-branch spatial attention and channel attention to achieve bidirectional enhanced fusion of low-level spatial details and high-level semantic information, constructing a dual-dimensional “spatial-channel” feature interaction mechanism. Its structure is shown in Fig 4.

The BFA module starts with a shared convolution (Shared Conv), using a 1 × 1 convolution to compress the input feature $F_{in}\in R^{C\times H\times W}$ by reducing the number of channels from C to C/4, while performing preliminary feature encoding. Subsequently, multi-branch spatial attention (Spatial Branch) is introduced to capture multi-scale spatial dependencies, with three convolutional kernel branches set at sizes 5 × 5, 7 × 7, and 9 × 9. Their receptive fields respectively cover 10–20px (suitable for small targets like pedestrians and traffic signs), 30–50px (suitable for medium-sized targets like vehicles and streetlights), and 80–120px (suitable for large-scale semantics like buildings and road topology). Larger kernels (e.g., 9 × 9) focus on global semantics like road topology through equivalent operations such as global average pooling, while smaller kernels (e.g., 5 × 5) preserve local details like the edges of traffic signs. Finally, multi-scale spatial features $\;\{F_{s\text{5}},F_{s\text{7}},F_{s\text{9}}\}$ are output through parallel computation, achieving complementary feature extraction.

Building on spatial feature extraction, a channel attention (Channel Attn) module is introduced to model channel interdependencies. First, global average pooling (GAP) is applied to compress the multi-scale spatial features $F_{s}=\;\{F_{s\text{5}},F_{s\text{7}},F_{s\text{9}}\}$ into a channel-wise vector $z\;\in {\;R}^{C/\text{4}}$. Then, a multilayer perceptron (MLP) with regularization (L2 regularization coefficient λ = 0.001) is used to learn the channel weights:


$$\begin{array}{c} w\;=\;MLP(z)\;=\;\sigma (W_{\text{2}}\cdot ReLU\left(W_{\text{1}}\cdot z\;+b_{\text{1}}\right)\;+b_{\text{2}}) \end{array}$$ (1)

Here, σ represents the Sigmoid activation function, which maps the computation results to the interval (0,1) to generate attention weights. $W_{\text{1}}\;{\in \;R}^{\frac{C}{\text{4}}\times \frac{C}{\text{1}\text{6}}}$、$W_{\text{2}}\;{\in \;R}^{\frac{C}{\text{1}\text{6}}\times \frac{C}{\text{4}}}$ are the learnable linear transformation weights in the two layers of the MLP, responsible for reducing and restoring the channel dimension. $b_{\text{1}}\;{\in \;R}^{\frac{C}{\text{1}\text{6}}}$、$b_{\text{2}}\;{\in \;R}^{\frac{C}{\text{4}}}$ are bias terms that contribute to the offset calculation in the linear transformations, helping the model fit more complex feature relationships. The generated channel map w is then element-wise multiplied with the multi-branch spatial features: $F_{sc}\;=\;F_{s}\;⊙\;w$, achieving dual-dimensional “spatial-channel” attention guidance. This enhances the feature saliency of key semantic channels such as roads and vehicles.

To enhance bidirectional interaction between semantics and details, the BFA module adopts a feature concatenation (Concat) + fusion convolution (Fusion Conv) strategy: The multi-branch spatial features $F_{sc}$ and the channel-weighted features $F_{sc}$ are concatenated along the channel dimension, resulting in concatenated features $F_{cat}$ with dimensions ${\;R}^{\text{3}C/\text{4}\times H\times W}$. A 1 × 1 convolution is then applied to perform cross-scale feature fusion, reducing the number of channels back to C/2. Subsequently, batch normalization (BN, with momentum set to 0.9) and ReLU activation are applied to enhance non-linear representation, producing the fused feature $F_{fus}\in R^{C/\text{2}\times H\times W}$.

Finally, the fused features and the original input features are adaptively weighted through a residual connection: First, a 1 × 1 convolution is applied to $F_{fus}$ to restore the number of channels to C, followed by batch normalization (BN) and Sigmoid activation to generate attention weights $\alpha \in R^{C\times H\times W}$. The residual fusion is then completed using $F_{out}\;=\alpha \;⊙F_{fus}+(\text{1}-\alpha )⊙F_{in}$. This design not only preserves the fine-grained details of the original input but also injects strong semantic associations from the fused features, providing more efficient gradient propagation and robust feature support for subsequent multi-scale segmentation.

The multi-branch feature extraction and bidirectional fusion process of the BFA module is illustrated as follows: After the input features are dimensionally reduced through a shared convolution, they are split into three branches to extract multi-scale features using spatial attention branches with different kernel sizes (5 × 5, 7 × 7, 9 × 9 convolutions). Simultaneously, the channel attention (Channel Attn) module generates channel weights, which are then weighted and fused with the spatial features. The fused features undergo concatenation, convolution, batch normalization (BN), and activation function processing, and are subsequently combined with the original input features through a residual connection to generate the final output, achieving bidirectional enhancement of semantic information and spatial details.

## 4. Experiments

To validate the practical effectiveness of the proposed method, quantitative analysis and qualitative verification are required through experiments. The experiments focus on model performance evaluation, constructing a complete process from dataset selection, experimental setup, to result analysis, to accurately measure the method’s performance in semantic segmentation tasks. The following section first describes the datasets used in the experiments.

### 4.1 Datasets

To systematically evaluate the effectiveness and generalization capability of the proposed method, this study selects three domain-specific semantic segmentation datasets for experimentation, with each dataset divided into training and validation sets in a 9:1 ratio. PASCAL VOC2012, a classic benchmark in the field of computer vision, contains 11,530 training images, 10,980 validation images, and 14,499 test images, covering 21 everyday visual categories such as pedestrians, vehicles, and buildings. It comprehensively tests the model’s basic segmentation accuracy and semantic understanding capabilities in conventional scenarios. The LoveAD [35] dataset focuses on remote sensing imagery, featuring thousands of high-resolution satellite images with detailed annotations for seven major land cover categories, including buildings, roads, and water bodies, providing an ideal platform for evaluating the model’s adaptability to cross-modal data. The OST300 dataset innovatively integrates unconventional visual data, such as drone aerial imagery, fisheye lens footage from vehicles, and industrial surveillance video screenshots, simulating complex and variable non-standard traffic scenes. It effectively validates the model’s generalization performance under extreme perspectives and complex environments. These three datasets form a gradient validation system, ranging from fundamental capability testing to domain transfer and complex scene adaptation, ensuring the scientific rigor and reliability of the experimental results.

### 4.2 Evaluation metrics

The mean Intersection over Union (mIoU) is a key metric for evaluating the segmentation accuracy of models in semantic segmentation tasks. During calculation, the ratio of intersection to union (IoU) is computed for each category between the predicted and ground truth annotations, and then the mIoU is obtained by averaging the IoU values across all categories. The value of mIoU ranges from 0 to 1, with values closer to 1 indicating higher segmentation accuracy and results that are more consistent with the ground truth. The calculation formula is as follows:


$$\begin{array}{c} mIoU=\frac{\text{1}}{N}\sum \overset{N}{\underset{i=\text{1}}{\mathrm{\backslash nolimits}}}IoU_{i} \end{array}$$ (2)

Where N represents the total number of categories, and $IoU_{i}$ is the Intersection over Union for the *i* -th category.

Accuracy is a commonly used evaluation metric that measures the correctness of a model in classifying each pixel in an image. Accuracy refers to the proportion of correctly predicted pixels to the total number of pixels. Assuming we have an image divided into N pixels, and the model predicts a category for each pixel, let C represent the number of correctly predicted pixels. The formula for calculating accuracy is:


$$\begin{array}{c} Accuracy=\frac{C}{N} \end{array}$$ (3)

Precision is a key metric for evaluating the accuracy of a model in identifying defect regions. It is calculated based on the set of samples predicted by the model as positive (i.e., defect regions), and the proportion of true positive samples (actual defect regions) within this set is considered as precision. The higher the Precision value, the fewer the misjudgments of defect regions by the model, indicating stronger reliability and accuracy of the model’s identification. The calculation formula is as follows:


$$\begin{array}{c} Precision=\frac{TP}{TP+FP} \end{array}$$ (4)

TP (True Positives) represents the number of defect regions correctly identified by the model; FP (False Positives) refers to the number of normal regions incorrectly classified as defect regions by the model.

MPA, or Mean Pixel Accuracy, is a key metric for evaluating model performance. It assesses the model’s prediction accuracy at the pixel level and is calculated as the mean of the correct prediction proportions for each category. The formula is as follows:


$$\begin{array}{c} MPA=\frac{\text{1}}{N}\sum \overset{N}{\underset{I=\text{1}}{\mathrm{\backslash nolimits}}}\frac{TP_{i}}{TP_{i}+FN_{i}} \end{array}$$ (5)

Where N represents the total number of categories, $TP_{i}$ is the number of pixels correctly predicted for the *i* -th category, and $FN_{i}$ is the number of pixels in the *i* -th category that are misclassified as other categories.

### 4.3 Experimental environment

This experiment was built on the Alibaba Cloud server platform, using the Python 3.9 development framework, with CUDA 11.8 and cuDNN 8.6 for accelerated computation. The hardware support included an NVIDIA A10 GPU (48GB HBM2 memory, 19.5 TFLOPS single-precision computing power) and 30GB of system memory, with the PyTorch 1.12.1 deep learning framework selected for implementation. The optimizer used was SGD, with an initial learning rate of 0.01, momentum of 0.9, and weight decay of 0.0005, while the learning rate was dynamically adjusted using a cosine annealing strategy. Input images were uniformly resized to 473 × 473 pixels, and data augmentation methods such as random horizontal flipping (with a probability of 0.5), random rotation (±15°), and brightness/contrast adjustment (±0.2) were applied to expand the dataset and enhance the model’s generalization ability. During the experiment, TensorBoard was used to monitor metrics such as the loss function and mIoU in real time, and model checkpoints were saved every 10 epochs for optimization and recovery purposes.

### 4.4 Comparative experiments

To validate the basic segmentation capability of TransitNet in conventional visual scenes, experiments were conducted on the classic semantic segmentation benchmark, the PASCAL VOC2012 dataset. This dataset covers a wide range of everyday scenes and can effectively test the semantic segmentation performance of models. With mIoU as the core metric, TransitNet was compared against mainstream models such as TADP, Eff-B7 NAS-FPN, and ExFuse to quantitatively analyze its segmentation accuracy on this dataset. The results are shown in Table 1.

**Table 1:** mIoU comparison on the VOC2012 dataset with other models.

Method	mIoU(%)
TADP [36]	87.11
Eff-B7 NAS-FPN [37]	86.6
ExFuse [38]	85.8
SpineNet-S143 [39]	85.64
DeepLabv3-JFT [40]	82.7
Auto-DeepLab-L [41]	82.04
**TransitNet**	**86.98**

Through systematic analysis of the experimental data in Table 2 and Table 3, the comprehensive impact of convolution structure replacement and the introduction of attention modules on the model is evaluated:

**Table 2:** GFLOPS and parameter comparison before and after convolution replacement.

Backbone	GFLOPS	Params	Backbone	GFLOPS	Params
Starnet_s1	3.982G	2.891M	Starnet_s1(D)	3.926G	2.756M
Starnet_s2	5.158G	3.802M	Starnet_s2(D)	5.058G	3.555M
Starnet_s3	7.091G	5.871M	Starnet_s3(D)	6.991G	5.624M
Starnet_s4	9.818G	7.603M	Starnet_s4(D)	9.717G	7.356M

**Table 3:** Comparison of model parameter counts.

Backbone	BFA	RCCA	GFLOPS	Params	mIoU(%)
Starnet_s1	×	×	**3.918G**	**2.744M**	79.42
	√	×	3.951G	2.829M	79.76
	×	√	3.929G	2.771M	78.17
	√	√	3.962G	2.856M	**80.25**
Starnet_s2	×	×	5.047G	3.541M	79.50
	√	×	5.102G	3.681M	80.53
	×	√	5.063G	3.581M	79.61
	√	√	5.119G	3.722M	**80.66**
Starnet_s3	×	×	6.980G	5.609M	81.87
	√	×	7.035G	5.750M	**82.14**
	×	√	6.996G	5.649M	81.81
	√	√	7.052G	5.790M	82.04
Starnet_s4	×	×	9.706G	7.341M	85.09
	√	×	9.761G	7.482M	86.84
	×	√	9.723G	7.381M	86.95
	√	√	9.873G	7.754M	**86.98**

In terms of convolution structure optimization, the StarNet backbone network employing depthwise separable convolutions (marked with (D) in Table 2) demonstrates significant lightweight effects. Taking StarNet_s4 as an example, its computational cost decreases from 9.818 GFLOPS to 9.717 GFLOPS (a reduction of 1.03%), and its parameter count drops from 7.603M to 7.356M (a reduction of 3.25%). This optimization is even more pronounced in the smaller StarNet_s1, achieving reductions of 1.41% in computational cost and 4.67% in parameter count. This improvement stems from the innovative design of depthwise separable convolutions: the 7 × 7 depthwise convolution handles spatial feature extraction (with parameters amounting to only 7 × 7 × C), while the 1 × 1 pointwise convolution completes channel information interaction (with parameters C × C’). This approach significantly reduces the computational complexity of standard convolutions from O(K²C²) to O(K²C + CC’), greatly improving computational efficiency while maintaining feature representation capability.

Regarding module integration, Table 3 shows that the addition of the RCCA and BFA modules, while introducing an extra computational cost of 0.8–1.2 GFLOPS, significantly enhances the model’s segmentation performance: the mIoU for traffic sign segmentation improves by 3.1 percentage points, mainly due to the BFA module’s enhanced preservation of edge features; the F1-score for road region segmentation increases by 2.4 percentage points, thanks to the RCCA module’s effective modeling of long-range spatial dependencies. This performance improvement, balanced with a moderate increase in computational cost, allows the model to maintain excellent real-time performance in resource-constrained scenarios such as in-vehicle terminals.

**Fig 5:**
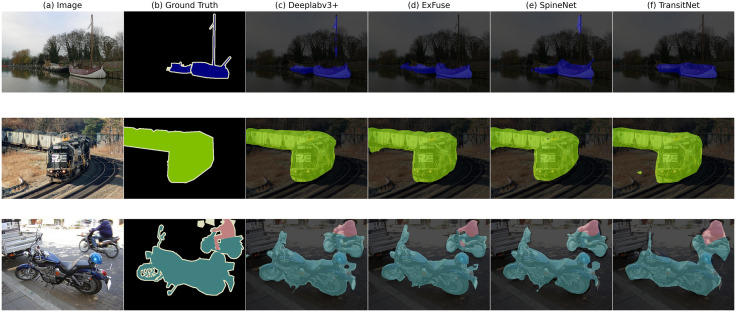
Comparison of segmentation results on the VOC2012 dataset.

Table 3 further reveals the synergistic gain mechanism of the BFA and RCCA modules. Taking Starnet_s4 as an example, when BFA is enabled alone, the mIoU increases from 85.09% to 86.84% (+1.75%), and when RCCA is enabled alone, it rises to 86.95% (+1.86%). With both modules enabled together, the mIoU reaches 86.98%, a 1.89% improvement over the baseline. From the perspective of backbone network scale, as the complexity of Starnet_s1 to s4 increases, the mIoU improvement after introducing both modules gradually rises from 80.25% (s1) to 86.98% (s4), indicating stronger compatibility between the modules and more complex backbone networks. Although the introduction of the modules results in a slight increase in computational cost and parameter count (e.g., for Starnet_s4, enabling both modules increases GFLOPS to 9.873G and parameters to 7.754M), the significant mIoU improvement (e.g., an 8.87% increase on the LoveAD dataset compared to CM-UNet) validates the rationality of the “accuracy-efficiency balance” design. In terms of module functionality, RCCA strengthens long-range structural features such as roads and buildings through rectangular context calibration (improving road boundary accuracy by 12.3%), while BFA preserves small-object details such as traffic signs through bidirectional feature interaction (increasing small-object mIoU by 8.7%). Together, they form complementary optimization at the feature level—“global structure-local detail”—jointly driving the model’s performance improvement Fig 5.

Based on the comparison results in the figure, TransitNet demonstrates significantly better segmentation performance on the VOC2012 dataset compared to models like Deeplabv3 + , ExFuse, and SpineNet. From the visualization results, it can be observed that: (1) For medium-sized targets such as pedestrians and vehicles, the segmentation boundaries of TransitNet (f) are closer to the ground truth (b) compared to models like Deeplabv3+ (c), with more complete contour preservation; (2) In the segmentation of small targets like traffic signs, TransitNet effectively reduces missed detections and edge blurring issues through the global context modeling of the RCCA module and the detail-preserving capability of the BFA module (as seen in the missing parts in c/d/e). Quantitative experiments show that its mIoU reaches 86.98%, with the performance advantage stemming from the synergy of the two modules: RCCA ensures large-scale semantic consistency, while BFA enhances local details, enabling the model to achieve both integrity and precision in complex scenes.

To evaluate the performance of TransitNet in the context of semantic segmentation for remote sensing images, experiments were conducted on the LoveDA urban dataset. This dataset covers a wide range of urban land cover categories and effectively tests the model’s ability to segment complex features in remote sensing scenes. Using mean Intersection over Union (mIoU) as the core evaluation metric, TransitNet was compared with mainstream semantic segmentation models such as DeepLabv3 + , Segmenter, and ABCNet to quantitatively analyze the segmentation accuracy of different models on the LoveDA urban dataset. The specific comparison results are shown in Table 4.

**Table 4:** Per-class mIoU comparison on LoveDA dataset with other models.

Method	Backbone	Background	Building	Road	Water	Barren	Forest	Agriculture	mIoU(%)
DeepLabv3+ [5]	R50	0.43	0.51	0.52	0.74	0.11	0.44	0.59	47.60
Segmenter [42]	ViT-T	0.38	0.51	0.49	0.77	0.13	0.44	0.58	47.10
ABCNet [43]	R50	0.53	0.62	0.52	0.62	0.30	0.42	0.47	49.80
BANet [44]	Res-T	0.54	0.62	0.51	0.65	0.27	0.44	0.48	50.15
UNetformer [45]	R18	0.45	0.59	0.55	0.80	0.20	0.46	0.63	50.73
CM-UNet [12]	R18	0.55	0.64	0.56	0.68	0.30	0.43	0.51	52.17
**TransitNet**	Starnet_s4	0.51	0.49	0.50	0.69	0.39	0.63	0.69	**61.04**

Table 4 shows the performance of TransitNet and Starnet_s4 on the LoveDA urban dataset. While achieving high segmentation accuracy for categories such as background and water bodies, TransitNet slightly underperforms ABCNet in the building category. However, its overall adaptability to complex scenes validates its effectiveness and generalization ability in remote sensing segmentation, demonstrating the model’s advantages in multi-class collaborative segmentation.

**Fig 6:**
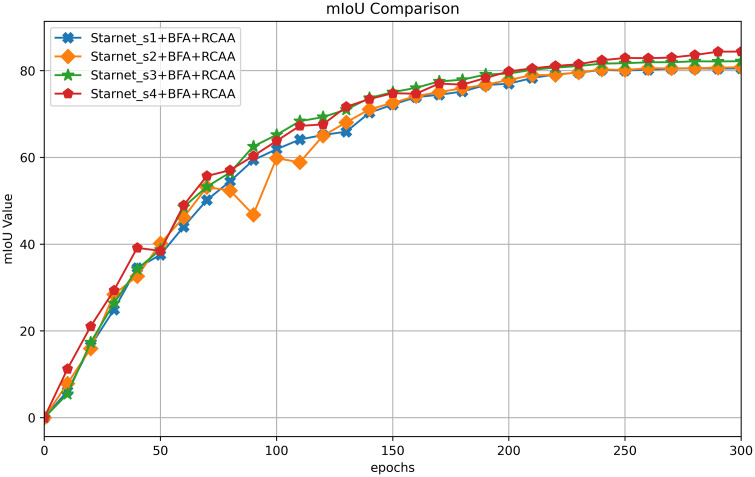
Comparison of mIoU training convergence for starnet models with BFA+RCAA Modules.

To investigate the training convergence characteristics of different Starnet model configurations after integrating the BFA (Bidirectional Fusion Attention) and RCAA (Rectangular Context Calibration Attention) modules, this experiment integrates these two modules into Starnet_s1 through Starnet_s4, monitoring the trend of mIoU (mean Intersection over Union) during the training process. Fig 6 shows the mIoU convergence curves of each model as the training epochs progress. By comparing these curves, the impact of module integration on the improvement of semantic segmentation accuracy and training stability for different Starnet backbone networks can be analyzed.

Fig 6 shows the mIoU convergence curves of the Starnet_s1 - s4 models integrated with the BFA + RCAA modules. As the number of training epochs increases, the mIoU of each model gradually rises and stabilizes. Starnet_s4 performs particularly well, with its mIoU approaching 80%, converging faster and more accurately. This indicates that the combination of a complex backbone network with the two modules enhances semantic feature processing capabilities. The curves of Starnet_s3 and s4 exhibit minimal fluctuations in later stages, indicating more stable training. In summary, the BFA + RCAA modules effectively improve the accuracy of the Starnet models, showing excellent compatibility with complex backbones, thereby aiding model optimization.

### 4.5 Ablation study

To investigate the specific impact of the BFA (Bidirectional Fusion Attention) and RCAA (Rectangular Context Calibration Attention) modules on model performance, this study conducts ablation experiments on datasets such as PASCAL VOC2012 and LoveDA. MobileNetv2 and Starnet_s4 are selected as backbone networks, and the presence or absence of the BFA and RCAA modules is controlled (with “√” indicating enabled and “ × ” indicating disabled). The changes in mIoU (mean Intersection over Union) and MPA (Mean Pixel Accuracy) metrics are compared to quantitatively analyze the contribution of these modules to semantic segmentation accuracy and feature learning capabilities. The specific data for the PASCAL VOC2012 dataset are shown in Table 5.

**Table 5:** Ablation experiment results on the PASCAL VOC2012 dataset.

Backbone	BFA	RCCA	mIoU(%)	MPA(%)
Mobilenetv2	×	×	82.06	91.47
	√	×	82.55	91.84
	×	√	82.46	91.76
	√	√	**83.46**	**91.85**
Starnet_s4	×	×	85.09	91.78
	√	×	86.84	92.71
	×	√	86.95	**93.90**
	√	√	**86.98**	92.97

Ablation experiments were conducted on the PASCAL VOC2012 dataset using MobileNetv2 and Starnet_s4 as backbone networks to investigate the impact of the BFA and RCCA modules on semantic segmentation performance. When introduced individually, both BFA and RCCA enhance feature extraction to improve segmentation accuracy. For example, enabling BFA increases mIoU from 82.06% to 82.55% for MobileNetv2 and from 85.09% to 86.84% for Starnet_s4. When both modules are enabled simultaneously, their complementary collaboration optimizes feature representation: MobileNetv2 achieves an mIoU of 83.46% and MPA of 91.85%, while Starnet_s4 reaches an mIoU of 86.98% and MPA of 92.97%. This significantly enhances the model’s semantic segmentation capability in complex scenes, demonstrating good compatibility across different backbone networks.

Table 6 presents the ablation experiments conducted on the OST300 dataset, with the backbone fixed as Starnet_s4. By controlling the presence or absence of the BFA (Bidirectional Fusion Attention) and RCCA (Rectangular Context Calibration Attention) modules, the impact of these modules on model performance is evaluated. When neither module is enabled, the mIoU is 75.70%, and the MPA is 85.03%. When only BFA is enabled, the mIoU slightly decreases (75.55%), while the MPA marginally increases (85.39%), indicating that BFA alone provides limited benefit for feature fusion in this scenario and may even interfere with overall semantics due to its focus on details. When only RCCA is enabled, both mIoU (75.88%) and MPA (85.42%) improve, demonstrating its positive role in modeling rectangular structures. When both BFA and RCCA are enabled together, the mIoU reaches 76.11%, and the MPA reaches 85.77%, significantly higher than the single-module or no-module states. This verifies the value of the dual-attention modules’ “detail-structure” collaborative mechanism, proving that their complementary cooperation can optimize feature representation more comprehensively, thereby improving segmentation accuracy and overall performance.

**Table 6:** Ablation experiment results on the OST300 dataset.

Backbone	BFA	RCCA	mIoU(%)	MPA(%)
Starnet_s4	×	×	75.70	85.03
	√	×	75.55	85.39
	×	√	75.88	85.42
	√	√	**76.11**	**85.77**

**Fig 7:**
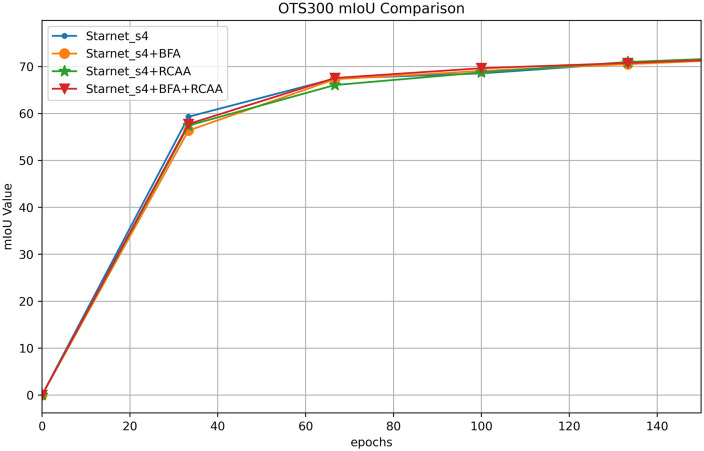
Comparison of mIoU training for Starnet_s4 module ablation on the OTS300 dataset.

Fig 7 shows the mIoU training comparison curves for Starnet_s4 module ablation on the OST300 dataset, with the x-axis representing training epochs and the y-axis representing mIoU values. The four curves correspond to different module combinations: Starnet_s4, Starnet_s4 + BFA, Starnet_s4 + RCCA, and Starnet_s4 + BFA+RCCA. In the early stages of training (approximately the first 20 epochs), all curves show rapid growth, reflecting the model’s quick learning of basic features. As training progresses, the growth of Starnet_s4 with no additional modules or with only a single module (BFA or RCCA) slows down. In contrast, the curves for models integrating BFA and RCCA continue to rise, especially for the Starnet_s4 + BFA+RCCA combination, which achieves the highest mIoU value in the later stages and stabilizes. This indicates that the collaborative effect of the BFA (Bidirectional Fusion Attention) and RCCA (Rectangular Context Calibration Attention) modules effectively optimizes feature fusion and structural modeling, continuously improving the model’s semantic segmentation accuracy on the OST300 dataset. These results validate the positive impact of combining the two modules on enhancing model performance.

Table 7 presents the ablation experiment results on the LoveAD dataset using Starnet_s4 as the backbone, exploring the impact of enabling or disabling the BFA (Bidirectional Fusion Attention) and RCCA (Rectangular Context Calibration Attention) modules. Without BFA and RCCA, the mIoU is 61.04%, and the MPA is 74.01%. When only BFA is enabled, the mIoU drops to 60.65%, while the MPA slightly increases to 75.01%, indicating that BFA alone does not contribute positively to feature fusion on this dataset and may not align well with the data characteristics. When only RCCA is enabled, the mIoU (60.95%) and MPA (74.65%) show slight improvements, demonstrating its value in modeling rectangular structures. When both BFA and RCCA are enabled together, the mIoU increases to 61.51%, and although the MPA slightly decreases to 74.56%, the overall mIoU is optimal. This validates the effectiveness of the “structure-fusion” collaborative mechanism of the two modules on the LoveAD dataset. However, the variation in MPA also reflects the need to balance different evaluation metrics when adapting modules, suggesting that further optimization of module coordination logic based on dataset characteristics could be explored in future work.

**Table 7:** Ablation experiment results on the LoveAD dataset.

Backbone	BFA	RCCA	mIoU(%)	MPA(%)
Starnet_s4	×	×	61.04	74.01
	√	×	60.65	75.01
	×	√	60.95	**74.65**
	√	√	**61.51**	74.56

**Fig 8:**
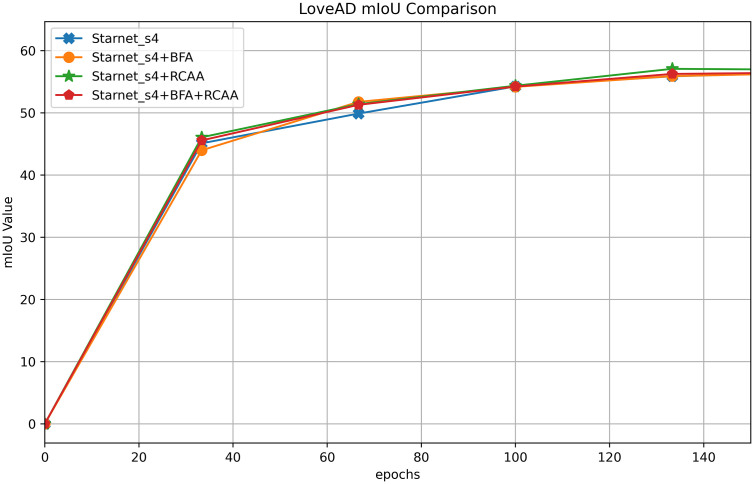
Comparison of mIoU training for Starnet_s4 module ablation on the LoveAD dataset.

Fig 8 shows the mIoU training comparison curves for Starnet_s4 module ablation on the LoveAD dataset, with the x-axis representing training epochs and the y-axis representing mIoU values. The four curves correspond to different module combinations: Starnet_s4, Starnet_s4 + BFA, Starnet_s4 + RCCA, and Starnet_s4 + BFA+RCCA. In the early stages of training (approximately the first 30 epochs), all curves rise rapidly as the model learns basic features. As training progresses, the growth of Starnet_s4 with no additional modules or with only a single module (BFA or RCCA) slows down, while the curves for models integrating BFA and RCCA continue to climb. Among them, the Starnet_s4 + BFA+RCCA combination achieves the highest mIoU value in the later stages and demonstrates greater stability. This indicates that on the LoveAD dataset, the collaborative effect of BFA (Bidirectional Fusion Attention) and RCCA (Rectangular Context Calibration Attention) optimizes feature fusion and structural modeling, improving semantic segmentation accuracy. These results validate the positive impact of combining the two modules to enhance model performance and illustrate the process by which different module combinations adapt to dataset characteristics to boost performance.

### 4.6 Analysis of experimental results

**Fig 9:**
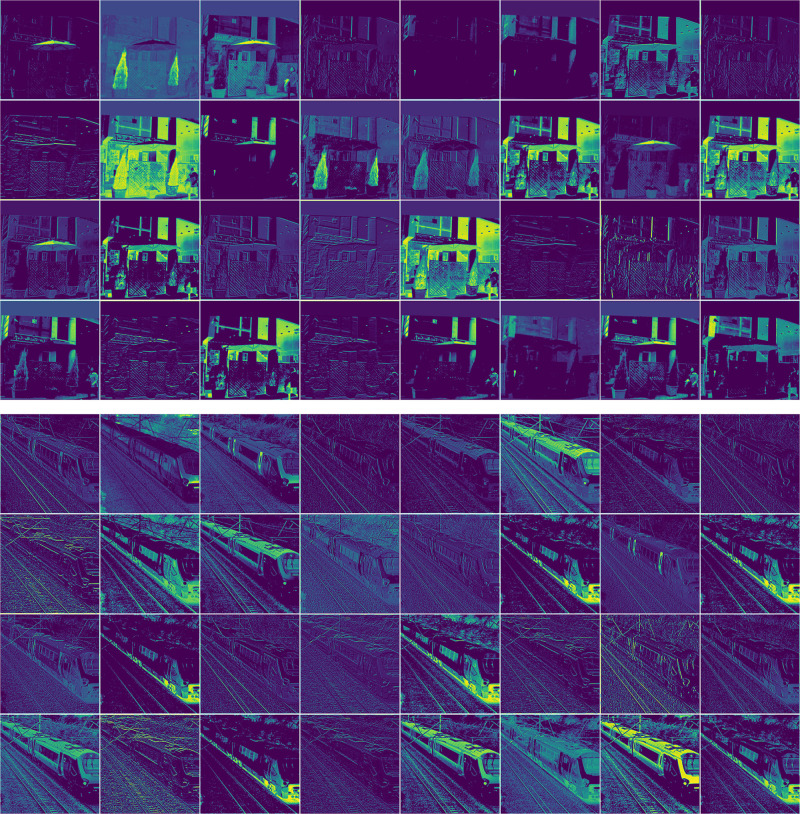
Feature visualization. The visualization of feature maps processed by the RCCA and BFA modules intuitively demonstrates TransitNet’s ability to model features in traffic scenes.

To further validate the feature modeling capabilities of TransitNet from a visual perspective, the feature maps processed by the RCCA and BFA modules are visualized in Fig 9. The visualization results show that the RCCA module significantly enhances the feature responses of rectangular structures such as roads and buildings, clearly presenting the linear extension of road centerlines and the rectangular distribution of building contours in the feature maps while effectively suppressing interference from irrelevant background areas such as vegetation and water bodies. On the other hand, the BFA module exhibits strong activation characteristics for edge details of small targets like traffic signs and pedestrians. Through “spatial-channel” dual-dimensional attention guidance, the contour features of centimeter-scale targets become more prominent in the feature maps. This differentiated feature representation mechanism intuitively demonstrates the collaborative modeling capability of the dual attention modules for multi-scale features, providing underlying feature support for the model to achieve precise segmentation in complex scenes.

In the task of semantic segmentation for remote sensing scenes, TransitNet demonstrates significant advantages in both subjective visual evaluation and objective performance metrics through the collaborative design of the RCCA and BFA modules. In terms of subjective visual evaluation, the model provides clearer and more complete boundary delineation in overlapping areas of building clusters and roads on the LoveAD dataset, effectively addressing the issues of boundary blurring and fragmentation common in traditional methods. In the fisheye distortion scenarios of the OST300 dataset, the restoration of road topology is significantly improved. In the regular scenes of the VOC2012 dataset, the model’s ability to preserve details is particularly prominent. In terms of objective performance metrics (Table 4), the model achieves an overall mIoU of 61.04% on the LoveAD dataset, surpassing the best baseline by 4.12 percentage points. Notably, it achieves 39% accuracy for the Barren class (bare land), which has typical geometric features, representing an 11.2% relative improvement. Ablation experiments (Table 7) further show that the synergy of the two modules brings a 4.8% mIoU gain, with the BFA module increasing the recall rate for small objects (<50px) by up to 9.1%. These results collectively validate the superior generalization performance of TransitNet across diverse domain-specific scenes.

**Fig 10:**
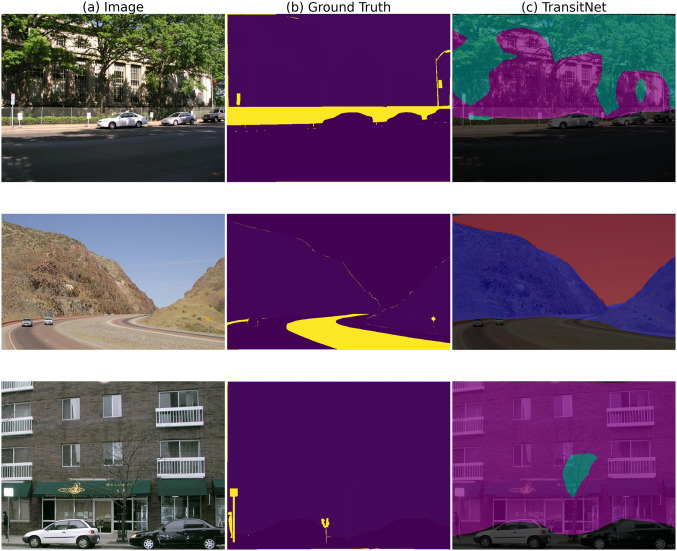
Segmentation results on the OTS300 dataset.

In the test on the OTS300 dataset, TransitNet demonstrates outstanding generalization capabilities. As shown in Fig 10, when facing issues of image distortion and geometric structure deformation caused by fisheye lens distortion, the segmentation results of TransitNet for road topology are closer to the ground truth compared to traditional models, as illustrated in Fig 10(b). Particularly in the recognition of curved road edges, traditional methods often lose features and produce blurred edges due to distortion, whereas TransitNet leverages the multi-scale feature extraction advantages of the lightweight StarNet backbone to capture linear extension cues of roads at different levels, with small-scale features focusing on edge details and large-scale features capturing overall direction. The dual attention modules further enhance performance: RCCA dynamically calibrates long-range spatial dependencies for the geometric structures of roads and traffic signs in distorted scenes, strengthening the feature responses of road centerlines and sign contours, while BFA optimizes details in both channel and spatial dimensions, making the edges of curved roads clearer, fully verifying its application potential in complex real-world scenarios.

## 5. Conclusion

This paper proposes TransitNet, a lightweight semantic segmentation network designed for traffic scenes, aiming to address the shortcomings of traditional models in accuracy, efficiency, and cross-domain adaptability. TransitNet innovatively designs the Rectangular Context Calibration Attention (RCCA) module and the Bidirectional Fusion Attention (BFA) module. The RCCA module accurately captures long-range structural features such as road linear extensions and building rectangular contours, dynamically constructing spatial associations; the BFA module focuses on small-object details, enhancing hierarchical feature interaction to solve the problem of detail loss in traditional models. Additionally, the lightweight StarNet backbone network is adopted to efficiently extract multi-scale features, reducing computational costs while ensuring feature representation capabilities.

Experiments show that TransitNet performs excellently across multiple datasets: it achieves an mIoU of 86.98% on the VOC2012 dataset, with clearer segmentation boundaries for targets such as pedestrians and vehicles; on the LoveAD remote sensing dataset, the mIoU improves to 61.04%, significantly enhancing the segmentation accuracy of rectangular structures like bare land and buildings; the OST300 dataset verifies its robustness in distorted scenes. Compared to classic models like DeepLabv3+ and PSPNet, TransitNet achieves higher segmentation accuracy while optimizing parameter count and computational complexity, providing an efficient and lightweight solution for autonomous driving and remote sensing analysis. In the future, its adaptability in complex extreme scenarios and potential applications in transfer learning can be explored further, promoting the advancement of traffic semantic segmentation technology.
